# LithiX Hertz Contact Lithotripsy Catheter for Calcium Modification: Insights From OCT and μFR Evaluation in Three Didactic Cases

**DOI:** 10.1016/j.jscai.2026.105504

**Published:** 2026-07-07

**Authors:** Ettore Ventura, Edoardo O. Genta, Stefano De Martini, Piero Montorsi, Stefano Galli

**Affiliations:** a Department of Interventional Cardiology, Centro Cardiologico Monzino, IRCCS, Milan, Italy; b Department of Clinical Sciences and Community Health, University of Milan, Centro Cardiologico Monzino, IRCCS, Milan, Italy

**Keywords:** calcium modification, case report, coronary physiology, intravascular lithotripsy, optical coherence tomography, percutaneous coronary intervention

## Abstract

The LithiX Hertz Contact Intravascular Lithotripsy System (Elixir Medical) is a new calcium fragmentation device that uses metal hemispheres on a semicompliant balloon to impart high contact stresses on calcified plaque while remaining inert on noncalcified tissue. We applied the Society for Cardiovascular Angiography & Interventions calcium-management algorithm to 3 consecutive complex calcified lesions, used optical coherence tomography as the intravascular imaging modality of choice in all cases, and integrated these findings with angiography-derived quantitative flow ratio for functional assessment before and after the procedure. Clinical follow-up was uneventful in all patients. The LithiX Hertz Contact Intravascular Lithotripsy System allowed effective calcium modification with a simplified, console-free setup and facilitated optimal stent expansion in complex and crossable lesions.

## Introduction

**Figure 1:**
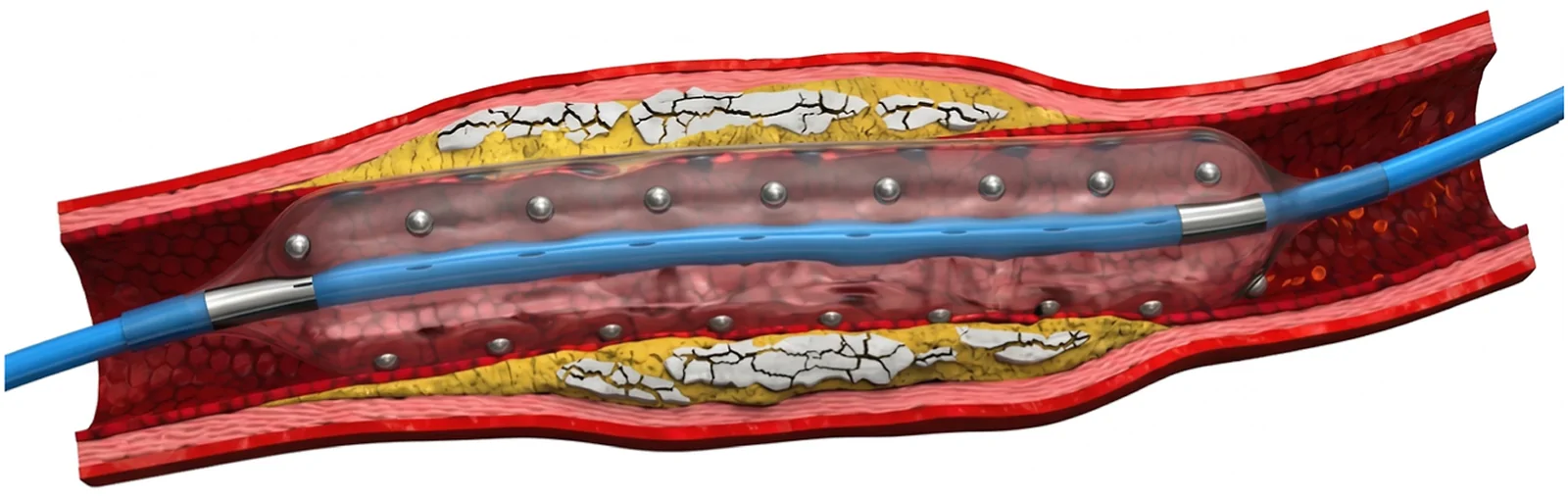
**The LithiX Hertz contact intravascular lithotripsy catheter.** Macroscopic view of the device demonstrating the semicompliant balloon equipped with multiple low-profile metallic hemispheres. IVL, intravascular lithotripsy.

Severely calcified coronary lesions remain a major cause of stent underexpansion; in fact, contemporary consensus statements recommend systematic intracoronary imaging to guide calcium modification strategies.^1^ The LithiX Hertz Contact Intravascular Lithotripsy (HC-IVL) System (Elixir Medical) is a mechanical catheter utilizing metallic hemispheres on a semicompliant balloon to impart high focal contact stresses on calcified plaque. Although the PINNACLE I trial demonstrated the macroscopic fragmentation efficacy of this device, the morphologic subtleties of focal stress and their physiological impact in real-world scenarios remain uncharacterized^2^ (Figure 1). We report 3 cases utilizing an algorithm-driven, imaging-first approach with optical coherence tomography (OCT) and angiography-derived quantitative flow ratio (μFR). For a complete view of the angiographic and imaging videos, please refer to the Supplementary material.

## Case 1

**Figure 2:**
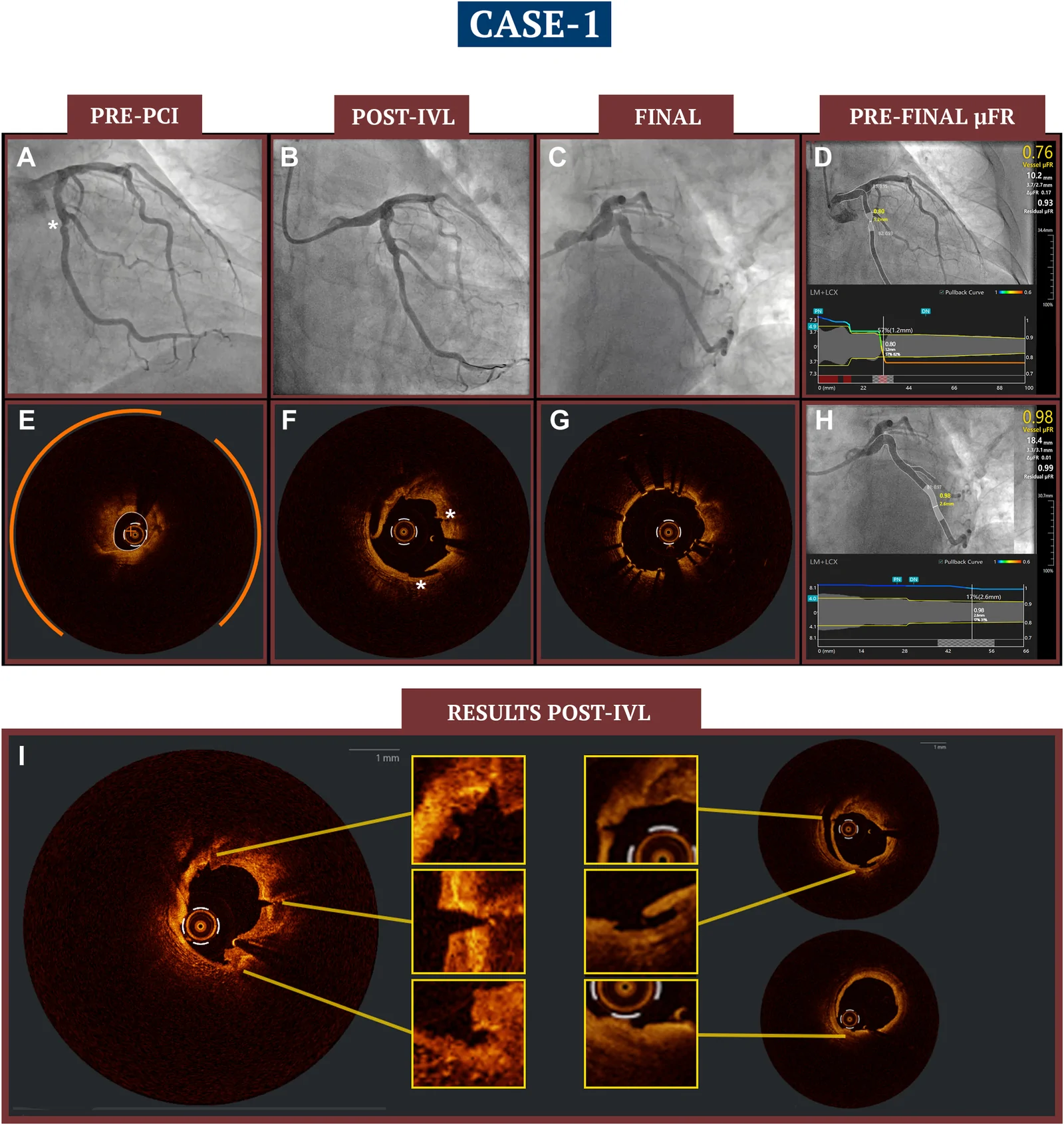
**Workflow of the first case.** Preprocedural coronary angiography of the target lesion (A), angiography immediately after Hertz contact intravascular lithotripsy (HC-IVL) (B), and final angiographic result after drug-eluting stent implantation (C). Angiography-derived quantitative flow ratio (μFR) before percutaneous coronary intervention (PCI) (D) and after stent implantation (H). Optical coherence tomography (OCT) phenotyping at baseline (E), immediately post HC-IVL (F), and at the final assessment (G). The magnified post HC-IVL OCT frame (I) highlights classical calcium fractures and hemispherical footprints.

**Figure 3:**
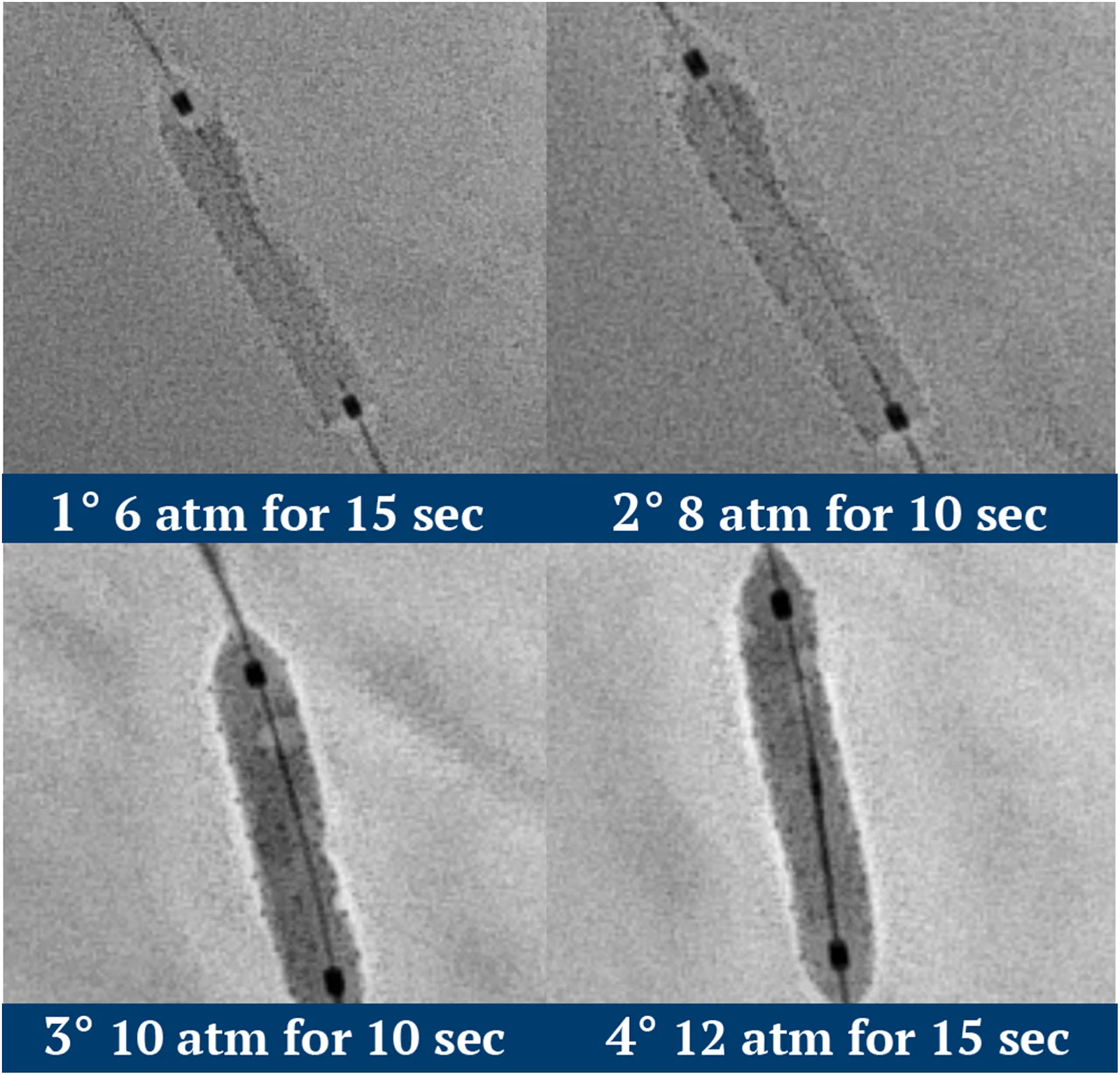
**LithiX Hertz contact lithotripsy stepwise inflation protocol.** atm, atmospheres.

An 81-year-old woman with multiple cardiovascular risk factors and chronic obstructive pulmonary disease presented with typical angina. Coronary computed tomography angiography (CCTA) demonstrated a severely calcified circumflex (LCx) stenosis. Radial angiography confirmed a focal, severely calcified mid-LCx lesion. Quantitative coronary angiography revealed a reference vessel diameter (RVD) of 3.7 mm, a diameter stenosis (DS) of 82%, and a lesion length of 10 mm. Angiography-derived μFR established the functional significance of the stenosis, with a vessel μFR of 0.76 and a perilesional ΔμFR of 0.17. In line with contemporary calcified-lesion algorithms, early intracoronary imaging was performed.^1^ An overview of the case workflow is shown in Figure 2. Baseline OCT demonstrated an eccentric calcific plaque with a minimum lumen diameter of 1.42 mm, minimum lumen area (MLA) of 1.60 mm^2^, maximum calcium arc of 300°, maximum calcium thickness of 1.1 mm, and calcium length of 6 mm. Given the complex calcium features, first-line plaque modification was undertaken with a HC-IVL 3.0 × 14 mm catheter using a stepwise inflation protocol at 6, 6 to 8, 8 to 10, and 10 to 12 atm for 10 to 15 seconds each (Figure 3). Post-HC-IVL OCT confirmed significant plaque modification with fractures (average width 0.35 mm, depth 0.58 mm) and characteristic hemispherical footprints, with the MLA increasing to 2.45 mm^2^. A DynamX 3.5 × 18 mm bioadaptor scaffold (Elixir Medical) was then implanted at 16 atm and proximally optimized with a 4.0 × 6 mm noncompliant (NC) balloon at 20 atm. Final OCT assessment documented a poststent MLA of 5.33 mm^2^, 104% expansion, and no edge dissections. The final vessel μFR improved to 0.98.

## Case 2

**Figure 4:**
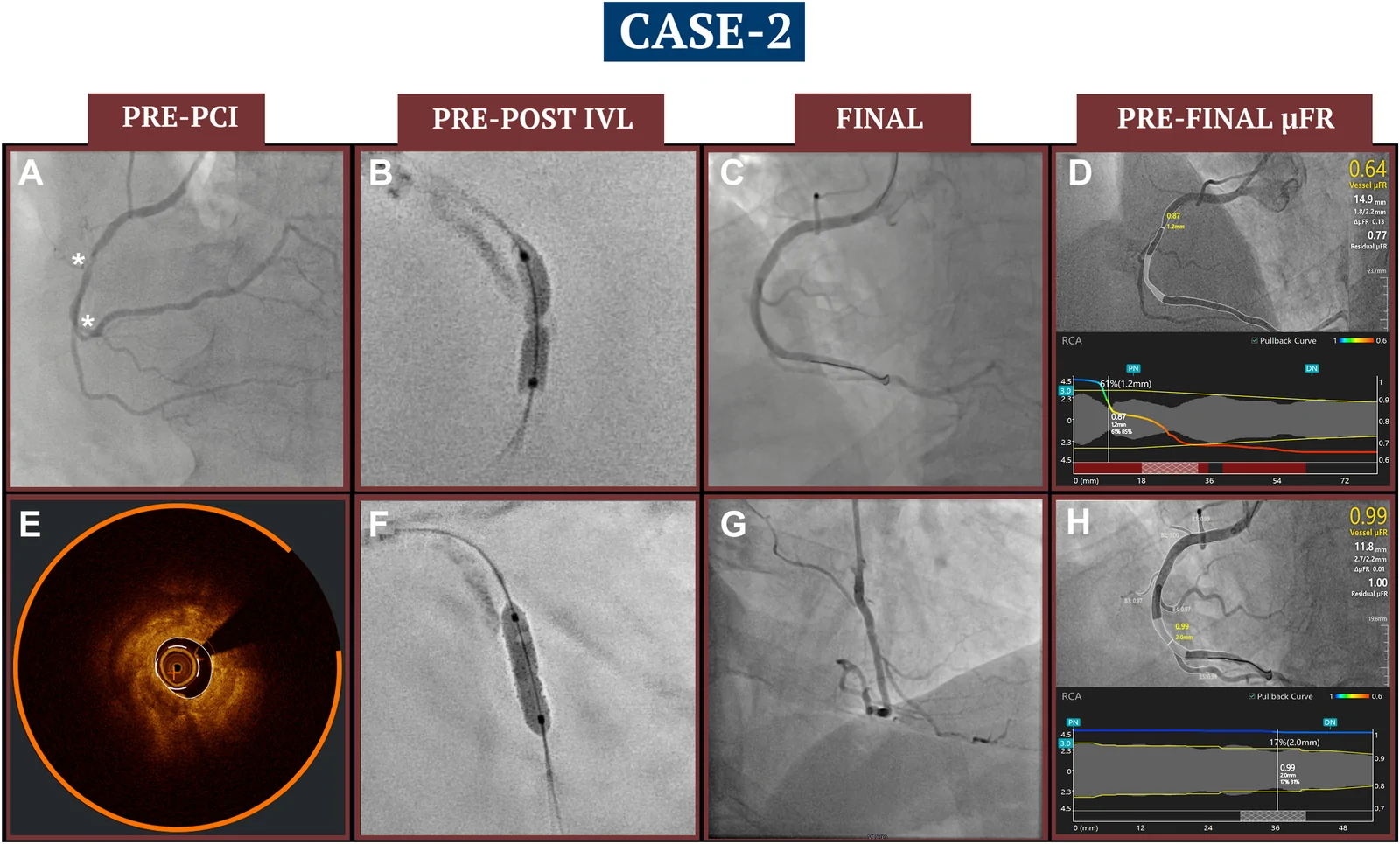
**Workflow ofCase 2.** Preprocedural coronary angiography (A) and optical coherence tomography baseline assessment (E) of the target lesion and angiography immediately after noncompliant balloon (B). ClearStent frames illustrate persistent underexpansion with a noncompliant balloon (B) and full expansion with the LithiX device across the calcified segment (F). Final angiographic result after drug-eluting stent (DES) implantation (C-G). Angiography-derived quantitative flow ratio (μFR) before percutaneous coronary intervention (PCI) (D) and after DES implantation (H).

A 77-year-old man with chronic kidney disease and atypical angina presented with runs of nonsustained ventricular tachycardia. CCTA demonstrated a markedly calcified right coronary artery stenosis. Angiography confirmed severe proximal-to-mid-right coronary artery calcific disease (DS 85%, RVD 2.3 mm, vessel μFR 0.64 with perilesional ΔμFR values of 0.18 and 0.13, respectively). The key elements of the case workflow are illustrated in Figure 4. Baseline OCT showed a heavily calcified plaque (arc 348°, thickness 1.5 mm, MLA 1.48 mm^2^). Because the lesion was tight and not crossable with HC-IVL 3.0 × 14 mm, stepwise predilatation was undertaken with semicompliant balloons 1.5 × 15 mm and 2.0 × 15 mm followed by an NC balloon 2.5 × 15 mm at high-pressure, which nevertheless remained underexpanded. With the aid of a 6F GuideLiner catheter extension (Teleflex), HC-IVL was then performed using a LithiX 3.0 × 14 mm catheter with multiple inflations at 6 to 8, 10, and 12 atm, achieving excellent balloon expansion at 12 atm. Three overlapping Onyx Frontier DES (Medtronic) were subsequently implanted at 12 to 16 atm and postdilated with a 2.75 × 20 mm NC balloon at 16 to 18 atm. Final residual stenosis was 0% with Thrombolysis in Myocardial Infarction grade 3 flow, and the vessel μFR improved to 0.99. Final OCT was omitted due to the patient’s chronic kidney disease and contrast-sparing requirements.

## Case 3

**Figure 5:**
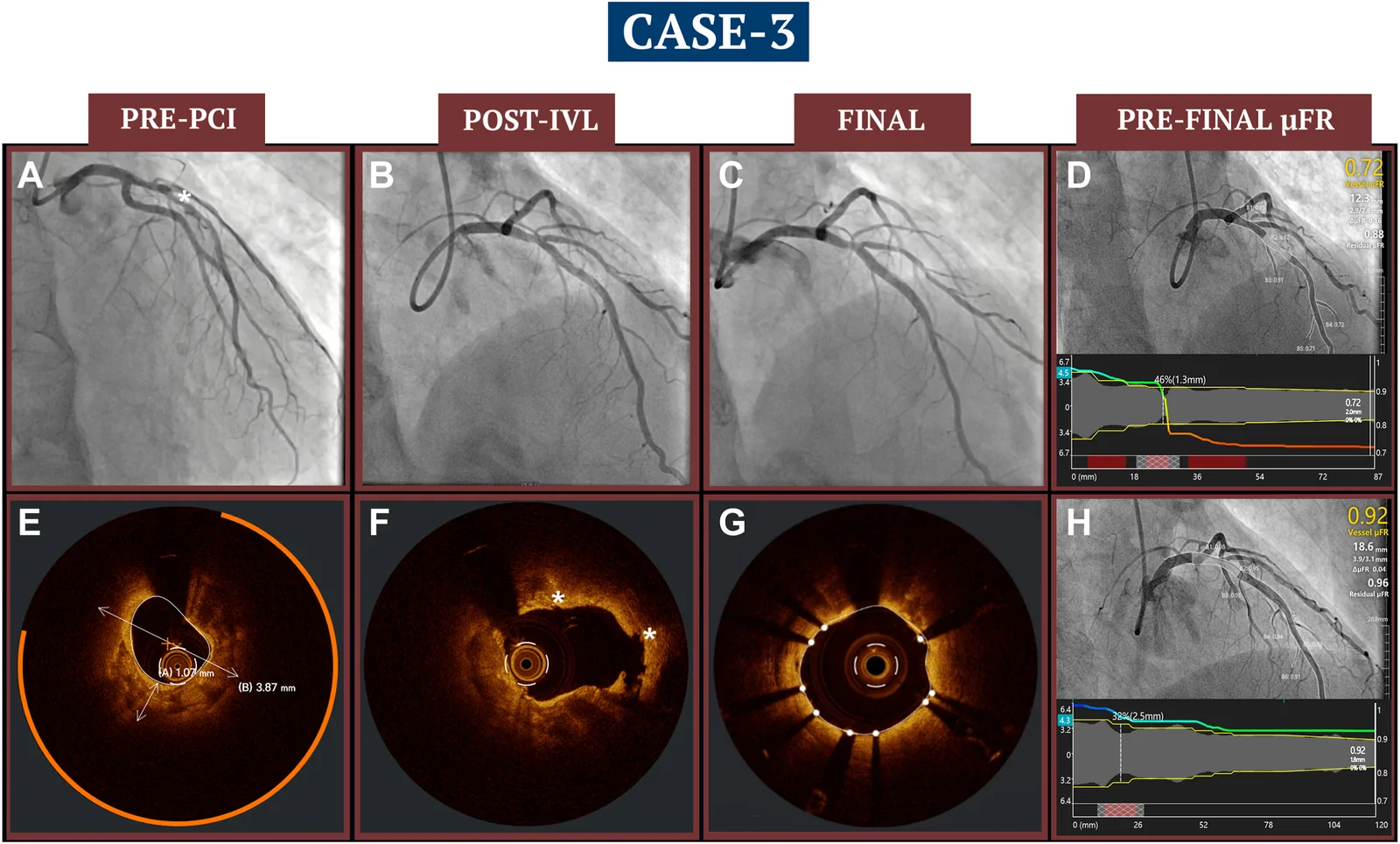
**Workflow of Case 3.** Preprocedural coronary angiography of the target lesion (A), angiography immediately after Hertz contact intravascular lithotripsy (HC-IVL) (B), and final angiographic result after Bioadaptor DES implantation (C). Angiography-derived quantitative flow ratio (μFR) before percutaneous coronary intervention (PCI) (D) and after DES implantation (H). Optical coherence tomography phenotyping at baseline (E), immediately after HC-IVL (F), and at the final assessment (G).

**Figure 6:**
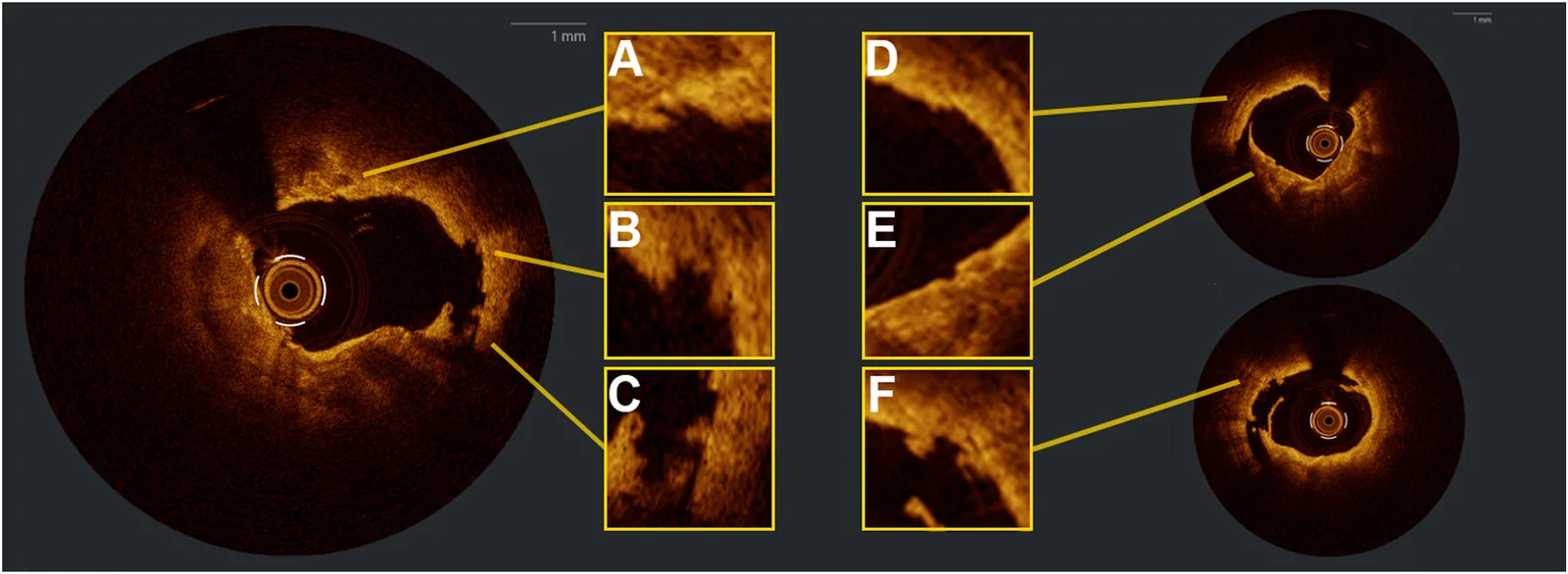
**Case 3 post-LithiXresults.** Post Hertz contact intravascular lithotripsy optical coherence tomography frames show calcium fractures in panels A, B, C, D, and F, whereas panel E displays a hemispherical footprint consistent with contact points of the Hertz contact intravascular lithotripsy device on calcified plaque.

A 74-year-old man with chronic obstructive pulmonary disease and multiple cardiovascular risk factors presented with exertional dyspnea. CCTA revealed severe left anterior descending artery and LCx calcified lesions. After staged LCx percutaneous coronary intervention, radial angiography revealed a long, severely calcified mid-left anterior descending artery stenosis (DS 70%, RVD 2.9 mm, vessel μFR 0.72, and perilesional ΔμFR of 0.16). The key elements of the case workflow are illustrated in Figure 5. Baseline OCT confirmed concentric tandem calcification (maximum arc 270°, thickness 0.8 mm, MLA 1.49 mm^2^). First-line modification proceeded with a 3.0 × 14 mm HC-IVL catheter using stepwise inflations up to 14 atm. Post-HC-IVL OCT demonstrated overt macro/microfractures (average width 0.42 mm, depth 0.65 mm) and distinct footprints, with the MLA increasing to 3.15 mm^2^ (Figure 6). A 3.0 × 28 mm DynamX bioadaptor scaffold was implanted at 14 atm and postdilated with a 3.5 × 15 mm NC balloon at 18 atm. Final OCT documented no edge dissections, a poststent MLA of 4.85 mm^2^, and a stent expansion of 103%. Functional reassessment showed a vessel μFR of 0.92.

## Discussion

This case series illustrates a standardized, imaging-first workflow for treating severely calcified coronary lesions using an HC-IVL system.^1^ By strictly adhering to the Society for Cardiovascular Angiography & Interventions calcium-management algorithm, we selected advanced plaque modification for targets characterized by “complex calcium features” on baseline OCT, which frequently predict the failure of conventional NC balloons. A comprehensive summary of the serial quantitative angiographic, OCT, and physiological measurements across the 3 cases is provided in Table 1. The LithiX device concentrates contact stress through focal metallic hemispheres, following the “hard on calcium, soft on tissue” principle to amplify stress at the plaque interface without requiring external consoles.^2^ The most notable morphological finding was the identification of hemispherical footprints on posttreatment OCT, alongside classical macrofractures. These imprints, observed even in the absence of overt full-thickness cracks, likely represent subsurface microcracking that compromises the structural integrity of the calcium plate. This phenomenon effectively lowers the yield stress of the plaque, as evidenced by the high final stent expansion indices achieved in our series. Clinical outcomes were uniformly favorable across the cohort. After the procedures, all patients remained asymptomatic with unchanged electrocardiograms and no elevation of myocardial necrosis biomarkers. Patients were discharged on the second postoperative day with a recommendation for 6-month dual antiplatelet therapy. At 1-month clinical follow-up, all patients remained asymptomatic with no documented major adverse cardiovascular events. Regarding the comparative efficacy, we acknowledge the potential role of high-pressure NC ballooning. However, the systematic failure of NC balloons in case 2, despite high-pressure inflation, highlights the limitations of purely mechanical dilation in resistant anatomy. By prioritizing advanced modification tools in lesions with high-risk imaging features per current consensus, we aim to minimize vessel trauma and optimize luminal gain.

**Table 1:** Serial angiographic, physiological, and OCT quantitative assessment

Parameter	Case 1 (LCx)	Case 2 (RCA)	Case 3 (LAD)
Baseline angiography (QCA)			
RVD, mm	3.7	2.3	2.9
Diameter stenosis, %	82	85	70
Lesion length, mm	10	30	13
Baseline physiology (μFR)			
Vessel μFR	0.76	0.64	0.72
Perilesional ΔμFR	0.17	0.31	0.16
Baseline OCT			
MLD, mm	1.42	1.31	1.35
MLA, mm^2^	1.6	1.48	1.49
Maximum calcium arc, °	300	348	270
Maximum calcium thickness, mm	1.1	1.5	0.8
Calcium length, mm	6	6	8
Post-HC-IVL OCT			
MLA, mm^2^	2.45	N/A^a^	3.15
Average fracture width, mm	0.35	N/A^a^	0.42
Average fracture depth, mm	0.58	N/A^a^	0.65
Final result OCT + μFR			
Final stent MLA, mm^2^	5.33	N/A^a^	4.85
Stent expansion, %	104	N/A^a^	103
Edge dissections	None	N/A^a^	None
Final vessel μFR	0.98	0.99	0.92

Summary of key measurements obtained by QCA, OCT, and physiology assessment at baseline, after HC-IVL, and following stent implantation.

HC-IVL, Hertz contact intravascular lithotripsy; LAD, left anterior descending artery; LCx, left circumflex artery; MLA, minimum lumen area; MLD, minimum lumen diameter; N/A, not available; OCT, optical coherence tomography; QCA, quantitative coronary angiography; RCA, right coronary artery; RVD, reference vessel diameter; μFR, Murray law-based quantitative flow ratio.

a Given the high contrast load and chronic kidney disease, no additional OCT runs were obtained.

## Conclusion

An algorithm-driven approach linking CCTA triage, OCT phenotyping, and HC-IVL enables targeted modification of complex coronary calcium. The identification of footprints provides a novel imaging marker of procedural success, correlating with physiological restoration and optimal stent expansion in a console-free setting.

## CRediT authorship contribution statement

**Ettore Ventura:** Writing – original draft, Data curation, Conceptualization. **Edoardo O. Genta:** Writing – review & editing, Investigation, Data curation, Conceptualization. **Stefano De Martini:** Writing – review & editing, Data curation, Conceptualization. **Piero Montorsi:** Writing – review & editing, Visualization, Conceptualization. **Stefano Galli:** Writing – review & editing, Writing – original draft, Visualization, Conceptualization.

## Declaration of competing interest

The authors declared no potential conflicts of interest with respect to the research, authorship, and/or publication of this article.

## Footnotes

To access the supplemental material accompanying this article, visit the online version of the *Journal of the Society for Cardiovascular Angiography & Interventions* at 10.1016/j.jscai.2026.105504.

## References

[1] Riley R.F., Henry T.D., Mahmud E. (2020). SCAI position statement on optimal percutaneous coronary interventional therapy for complex coronary artery disease. Catheter Cardiovasc Interv.

[2] Verheye S., Ferdinande B., Paradies V. (2026). Hertz contact intravascular lithotripsy for calcified coronary artery disease: the PINNACLE-I trial. EuroIntervention.

